# Parasite genomics—Time to think bigger

**DOI:** 10.1371/journal.pntd.0005463

**Published:** 2017-04-20

**Authors:** Carlos Talavera-López, Björn Andersson

**Affiliations:** Department of Cell and Molecular Biology, Karolinska Institutet, Berzelius väg 35 SE, Stockholm, Sweden; University of Washington, UNITED STATES

It has been more than 20 years since genomics approaches began to be applied to the problem of tropical neglected parasitic diseases in the form of pilot genome sequencing projects. The *Plasmodium falciparum* genome sequence was published in 2002 [1] as the result of an international collaborative effort, and the first reference genome sequences of *Trypanosoma cruzi*, *Trypanosoma brucei* and *Leishmania major* were published in 2005 [2]. Today, most medically relevant pathogenic protozoan have been the subject of genome sequencing projects.

The initial benefit of many sequencing projects was to boost the field of parasite molecular biology. With most genes of these organisms available for study, a large number of new functional genomics studies with focus on drug resistance, drug target identification and gene evolution could be undertaken in parallel, which has greatly increased activity in these fields [3]. As a result, the knowledge of parasite biology is increasing, and there are many promising approaches for the identification of new candidates for chemotherapeutic agents and vaccines.

The introduction of next generation sequencing technologies has now provided multiple new ways to utilize genomics for the study of parasitic diseases [4] and the integration with other types of large-scale biological data. These include comparative genome sequencing, transcriptomics, proteomics, metabolomics and epigenetics [5] of the parasite, as well as multiple aspects of the host biology. With these approaches, specific biological questions, such as drug resistance, epidemiology, genetic exchange, immune evasion mechanisms, gene function and others can be addressed at a large scale using integrative–omics approaches. In the case of human and many other pathogens such as *Plasmodium* parasites, many such studies have been undertaken and are in progress [6]. For the other neglected diseases, mostly smaller-scale pilot efforts have thus far been carried out [7].

There are several reasons why this is the case and some of these will be discussed below. For certain organisms, progress has been limited by the lack of a complete and reliable reference genome sequence. As a general rule, the greater the repeat content and complexity of the genome, the lower the quality of the initial genome sequence, due to the impossible task of assembling the repetitive regions using short sequencing reads. An example of this is *T*. *cruzi*, where 38% of the original reference genome sequence, containing thousands of biologically important surface molecule genes, was either missing or misassembled. The use of new long-read sequencing techniques have begun to resolve repetitive regions, resulting in vastly improved genome sequences for other species [8].

There are also other aspects of parasite biology that are relatively unexplored for many parasites and much basic research is needed before pathogenicity issues can be addressed effectively. These include a large proportion of genes where the function is unknown, unclear understanding of the parasite population structure, unresolved issues regarding the extent of genetic exchange, and even missing data on many of the basics of the mechanisms involved in disease causation. While many of these issues are perfect cases for the application of integrative–omics approaches, additional work is also needed for a complete characterisation of individual targets and gene families. The extent to which this applies varies depending on the biological complexity of certain parasites, the technological challenges and the resources (or lack thereof) that have been available to researches in the past.

Sampling is a very important—and often difficult—aspect for the study of many parasitic diseases. While there are many collections available in many cases, these are often not collected in a standardized way, they possess limited metadata and very often the sampling strategy does not have a specific biological question in mind. In order to address a particular biological question, large sample collections are often required to provide enough statistical support and the sampling strategy must be clear with stringent criteria for the characteristics of each sample category, as it has been clearly stated in other fields [9]. Sampling often requires field studies with visits to remote locations that are logistically complex and very costly. In addition, some of the sampled parasites, such as *Leishmania*, *T*. *cruzi*, *T*. *rangeli*, among other, cannot be studied without growth in culture and even cloning through limiting dilution. This introduces a level of uncertainty, as parasites may change in culture, and the clones that dominate in culture may not be the dominant ones in the host.

Single-cell genome and transcriptome sequencing has recently been developed [10], and this could potentially be a solution to this problem. Indeed, these studies have helped to expose a more accurate picture of the host-pathogen interactions in simple microbes [11], and they can be expanded to study more complex unicellular protozoan parasites while integrating different types of data [12]. While the cost of carrying out integrative–omics studies is steadily dropping, large scale studies are still expensive from the perspective of the field of neglected diseases, where research grants are usually limited. Notwithstanding, small to medium-scale studies, using dozens to hundreds of samples, can now be carried out using standard-level research grants aided by the massive capacity of modern high-throughput equipment.

Many questions will require the collection of host data, such as the full characterisation of host and/or vector immune responses with high dimensional flow cytometry [13], host microbiome profiling and even host genomic data [14], which will drastically increase the overall cost of the study.

This will need to be solved by a continuing reduction of technical costs, including data production and analysis, and—hopefully—increased funding form private and public agencies for properly designed–omics projects in neglected diseases.

As evidenced by the solutions to long-standing problems mentioned above and the progress made for other organisms, we are now in position to address many biological and medical issues for many neglected parasitic diseases. This advances will translate into applied studies for the development of vaccines and drug targets, which are heavily needed among susceptible populations.

This will require large collaborative efforts involving different areas of expertise, like epidemiology, parasite molecular biology, computational biologists, and big data analyst. Future studies will require the collection of thousands of samples, the production of different–omics data from parasites and hosts, as well as careful validation and follow-up of each finding using *in vitro* and animal model studies.

We can now transition from small pilot studies with unclear results to well-designed large studies with the aim to solve the important, long standing biological and medical questions for these neglected diseases.
